# A global biophysical typology of mangroves and its relevance for ecosystem structure and deforestation

**DOI:** 10.1038/s41598-020-71194-5

**Published:** 2020-09-04

**Authors:** Thomas A. Worthington, Philine S. E. zu Ermgassen, Daniel A. Friess, Ken W. Krauss, Catherine E. Lovelock, Julia Thorley, Rick Tingey, Colin D. Woodroffe, Pete Bunting, Nicole Cormier, David Lagomasino, Richard Lucas, Nicholas J. Murray, William J. Sutherland, Mark Spalding

**Affiliations:** 1 Conservation Science Group, Department of Zoology, University of Cambridge, Cambridge, CB2 3QZ UK; 2 Global Change Group, School of Geosciences, Grant Institute, Kings Buildings, University of Edinburgh, Edinburgh, EH9 3FE UK; 3 Department of Geography, National University of Singapore, 1 Arts Link, Singapore, 117570 Singapore; 4 U.S. Geological Survey, Wetland and Aquatic Research Center, 700 Cajundome Blvd, Lafayette, LA 70506 USA; 5 School of Biological Sciences, University of Queensland, St. Lucia, QLD 4072 Australia; 6 Independent GIS Consultant, Penzance, UK; 7 Spatial Support Systems, LLC, Cottonwood Heights, UT 84121 USA; 8 School of Earth Atmospheric and Life Sciences, University of Wollongong, Wollongong, NSW 2522 Australia; 9 Department of Geography and Earth Sciences, Aberystwyth University, Aberystwyth, Wales UK; 10 Department of Earth and Environmental Sciences, Macquarie University, Level 4, 12 Wally’s Walk, Sydney, NSW 2109 Australia; 11 Department of Coastal Studies, East Carolina University, Wanchese, NC 27981 USA; 12 Biospheric Sciences Laboratory, NASA Goddard Space Flight Center, Greenbelt, MD 20771 USA; 13 College of Science and Engineering, James Cook University, Townsville, QLD 4811 Australia; 14 The Nature Conservancy, c/o Department of Physical, Earth, and Environmental Sciences, University of Siena, Pian dei Mantellini, 53100 Siena, Italy

**Keywords:** Wetlands ecology, Biogeography

## Abstract

Mangrove forests provide many ecosystem services but are among the world’s most threatened ecosystems. Mangroves vary substantially according to their geomorphic and sedimentary setting; while several conceptual frameworks describe these settings, their spatial distribution has not been quantified. Here, we present a new global mangrove biophysical typology and show that, based on their 2016 extent, 40.5% (54,972 km^2^) of mangrove systems were deltaic, 27.5% (37,411 km^2^) were estuarine and 21.0% (28,493 km^2^) were open coast, with lagoonal mangroves the least abundant (11.0%, 14,993 km^2^). Mangroves were also classified based on their sedimentary setting, with carbonate mangroves being less abundant than terrigenous, representing just 9.6% of global coverage. Our typology provides a basis for future research to incorporate geomorphic and sedimentary setting in analyses. We present two examples of such applications. Firstly, based on change in extent between 1996 and 2016, we show while all types exhibited considerable declines in area, losses of lagoonal mangroves (− 6.9%) were nearly twice that of other types. Secondly, we quantify differences in aboveground biomass between mangroves of different types, with it being significantly lower in lagoonal mangroves. Overall, our biophysical typology provides a baseline for assessing restoration potential and for quantifying mangrove ecosystem service provision.

## Introduction

Mangrove forests provide valuable ecosystem functions and services including carbon storage, coastal protection, fisheries enhancement and tourism^1–4^; however, large declines in global mangrove area have historically been estimated^5^. Recent high-resolution assessments of mangrove change suggest considerable slowing of losses^6^, likely driven by growing wealth, increasing clarity of ownership, national efforts to sustainably manage forest estates and increased awareness of the ecosystem services they provide^7^.

At a global scale, mangroves are of considerable value to humans^8^ yet it is also recognised that the value derived from mangroves varies geographically and that this variability is as yet poorly quantified^9^. Mangroves show substantial geographic variation in structure, height^10^, and species diversity^11^, driven by factors such as climate, tidal amplitude and particularly geomorphic setting. These factors, in turn, can also influence variability in ecosystem functions and services such as carbon storage^12–16^, coastal protection^17^ and fisheries^18^.

Despite the importance of the geomorphic setting of mangroves in determining their ecosystem service delivery^12^, their relative risk under future climate change and sea level rise^19^ and in influencing optimal restoration actions^20^, many recent global analyses have assumed a spatial uniformity of mangrove forests^9,21^. This has, in part, been determined by the binary (presence/absence) nature of previously available global mangrove extent maps^11,22,23^. By contrast, recent efforts to quantify the variability in mangrove soil carbon have illustrated the utility of applying broad coastal geomorphic settings to explain levels of ecosystem service delivery^13–15^. However, until now a mangrove-specific global biophysical typology of geomorphic setting has not been generated. Such information would allow for tailored conservation and management strategies to be developed to protect ecosystem services provided by mangroves^24^ and determine appropriate restoration actions^20^.

Here, we present a global-scale, mangrove specific, biophysical typology that integrates the main drivers of spatial heterogeneity of mangrove ecosystems into mappable units. The biophysical typology was developed by reviewing existing, largely qualitative, classifications and applying a model of key spatial attributes that could be mapped consistently at the global scale. The biophysical typology was applied to maps of global mangrove extent generated by Global Mangrove Watch (GMW)^25^ and is not itself a predictor of mangrove presence or absence. This typology provides a framework for future analyses, allowing for better incorporation of the spatial heterogeneity of geomorphic and sedimentary setting. We provide two examples of such analyses: firstly, quantifying how mangrove extent change over the period 1996 to 2016 varied between different mangrove types; and secondly showing the potential application of our typology in informing global analyses of ecosystem structure using a dataset of mangrove above-ground biomass^10^.

## Results and discussion

### Global distribution of mangrove types

We sought to create a broad-scale biophysical typology that was parsimonious with existing theoretical classifications^12,26–29^, with our types, deltaic, estuarine, lagoonal, and open coast mangroves, comparable to previous typological classes (Table 1). Our efforts represent the first attempt to map a mangrove biophysical typology beyond individual case study areas. To map the biophysical typology, we developed a map of coastal embayments and used a machine-learning classifier to assign each embayment with a type through reference to ten environmental covariates. The biophysical typology was framed around three of the macroscale groupings defined by Woodroffe and colleagues^29^, and Twilley and Rivera-Monroy’s^28^ ‘geomorphic types’. In addition, we derived an ‘open coast’ type that incorporates several of the divisions in other typologies (Table 1), such as drowned bedrock valleys^26^ and carbonate mangroves found on oceanic islands^28^. The four mangrove types represent macroscale units with a resolution of kilometres^30^. Open coast and lagoonal mangroves were also assigned a second-tier sedimentary type, as either terrigenous (i.e. dominated by minerogenic sedimentation from terrestrial sources), or carbonate (i.e. dominated by calcareous sedimentation), based on sediment supply, and tidal energy^20^. Full definitions of the types are given in Supplementary Section 1.

**Table 1:** A summary of existing mangrove typologies illustrating the relationship between previously described mangrove types and the one developed and mapped in this study. Where GEO refers to geomorphic setting, and SED refers to sedimentary setting.

This Typology			Thom^26^	Woodroffe^27^	Twilley and Rivera-Monroy^28^	Balke and Friess^20^	Woodroffe and colleagues^29^
SED	GEO	Brief Definition of Geomorphic Setting	GEO/SED	GEO/SED	GEO	SED	GEO
Terrigenous	Deltaic	Shoreline protuberance typified by a wide fan-shaped alluvial plain derived from large volumes of river transported sediment	River-dominated allochthonous	River-dominated	Delta	Minerogenic	Delta
	Estuarine	Funnel shaped main channel with bidirectional tidal flows, characterised by large catchment area and high precipitation input	Tide-dominated allochthonous	Tide-dominated	Estuary		Tidal estuary
	Lagoonal	Shallow coastal waterbody, intermittently separated from ocean inputs. Usually formed parallel to the shore	Wave-dominated barrier lagoon	Wave-dominated	Lagoon		Lagoon
	Open coast	Sheltered embayments such as drowned bedrock valleys	Drowned bedrock valley	Drowned bedrock valley			
Carbonate	Lagoonal	See above	Sand/shingle barrier	Carbonate settings	Lagoon	Organogenic	Lagoon
	Open coast	Sheltered environments on oceanic islands behind coral reefs and carbonate banks	Low-energy coast		Oceanic islands		Carbonate reef

We used the most recently available high-resolution mangrove presence/absence time-series to map the biophysical typology and enable spatially explicit estimates of change in mangrove type. The GMW generated a 2010 baseline of mangrove extent^25^ using a combination of USGS Landsat and Japan Aerospace Exploration Agency (JAXA) Advanced Land Observing Satellite (ALOS) Phased Array L-band Synthetic Aperture Radar (PALSAR) data. Change was then mapped from the 2010 baseline using JAXA’s Japanese Earth Resources Satellite (JERS-1) (1992–1998; nominally for 1996), ALOS PALSAR (2007–2009) and ALOS-2 PALSAR-2 (2015–2016). To map the mangrove biophysical typology, we merged the GMW 1996, 2007, 2010 and 2016 time steps, to form a 20-year maximal mangrove extent of 145,595 km^2^. This total was split into 4,318 individual patches ranging in extent from 0.0005 to 6,517 km^2^. Within this maximal mangrove extent, approximately 40% (58,681 km^2^) of the world’s mangrove forest were confined to just 84 river deltas, with estuarine mangroves covering the next greatest area (n = 961 patches; 39,448 km^2^). These two dominant types can form large individual extents of mangrove where accretion of fluvially transported terrigenous sediment^29,31,32^ allows opportunistic colonization by mangroves^33–35^. Open coast mangroves covered an area of 30,586 km^2^ and were by far the most numerous unit type (n = 2,639). Open coast mangroves were prevalent in areas with limited freshwater and terrigenous sediment inputs, such as the Middle East and the Pacific Islands^29^. Lagoons were largely restricted to high wave energy coasts; conditions that limit the potential mangrove establishment^27^. This combination of factors helps to explain the minimal global coverage of lagoonal mangroves (n = 634; 16,880 km^2^).

**Figure 1:**
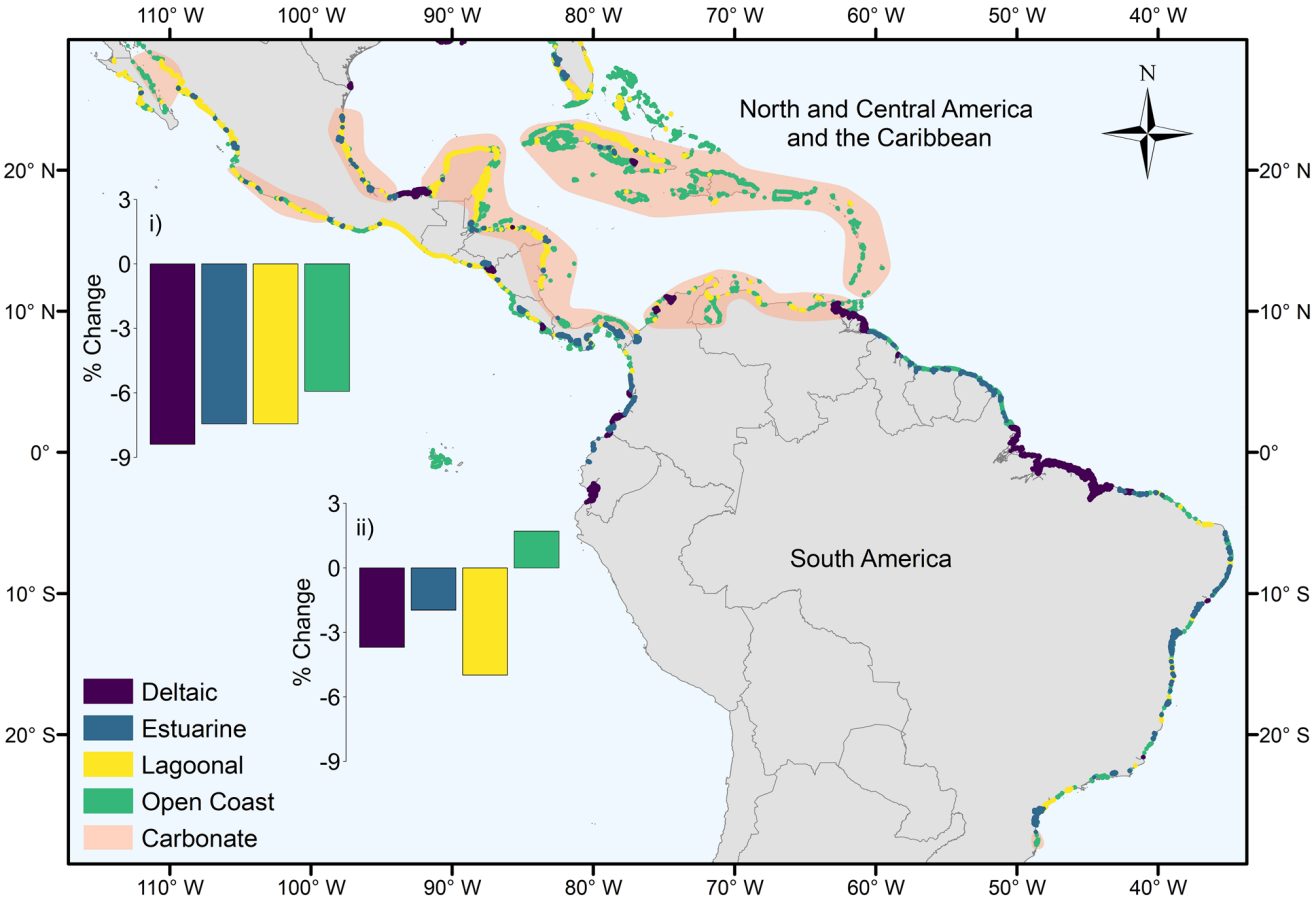
Distribution of deltaic, estuarine, lagoonal and open coast mangrove types, and approximate extent of carbonate sedimentary settings in the (i) North and Central America and the Caribbean and (ii) South America regions. Bar charts represent the percentage change in area of the different types between 1996 and 2016 at the regional scale. Adapted from Worthington and Spalding^38^. The map was generated in ArcGIS Desktop version 10.6 software (https://desktop.arcgis.com/en/).

**Figure 2:**
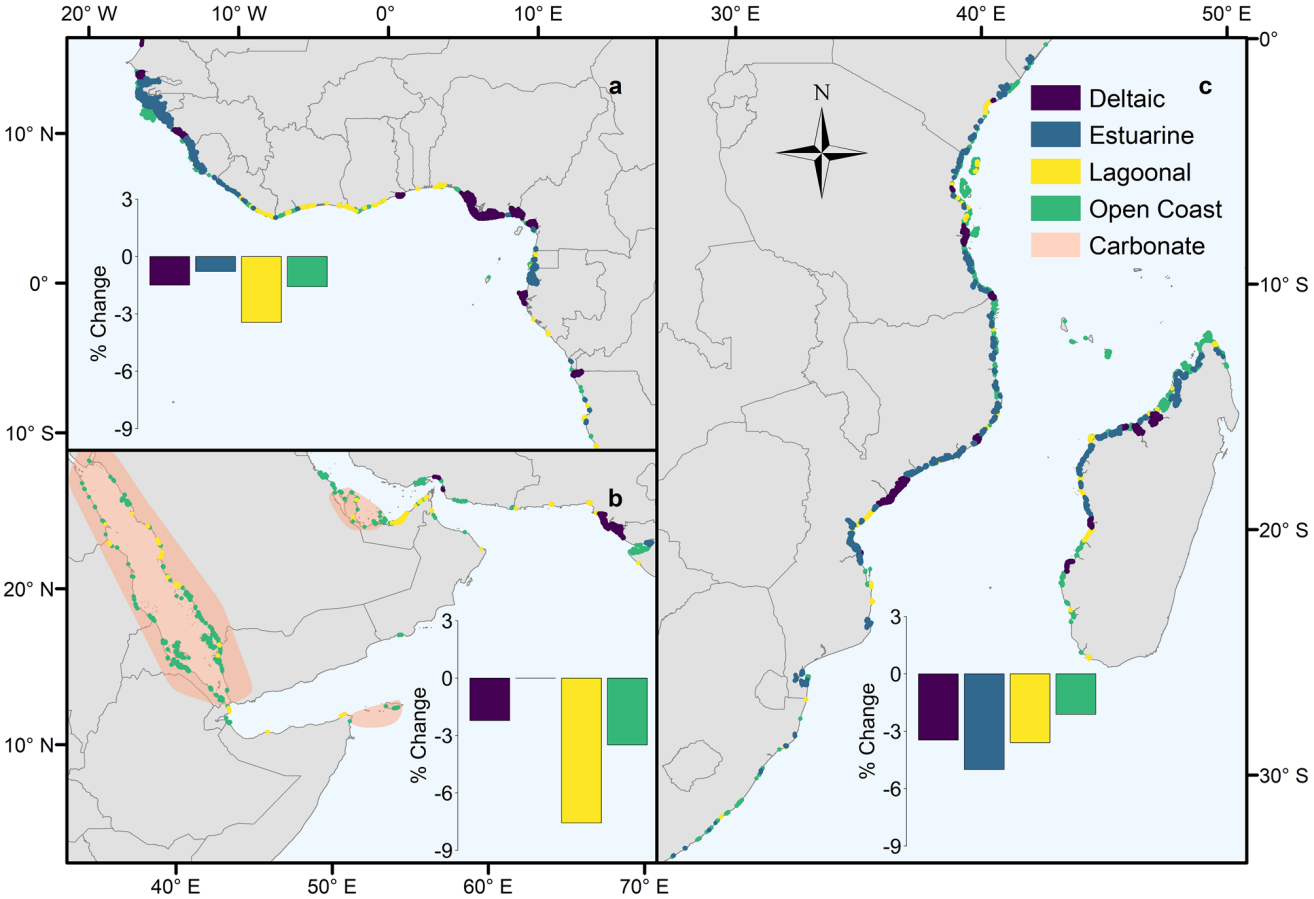
Distribution of deltaic, estuarine, lagoonal and open coast mangrove types, and approximate extent of carbonate sedimentary settings in the (**a**) West and Central Africa, (**b**) Middle East and (**c**) East and Southern Africa regions. Bar charts represent the percentage change in area of the different types between 1996 and 2016 at the regional scale. Adapted from Worthington and Spalding^38^. The map was generated in ArcGIS Desktop version 10.6 software (https://desktop.arcgis.com/en/).

**Figure 3:**
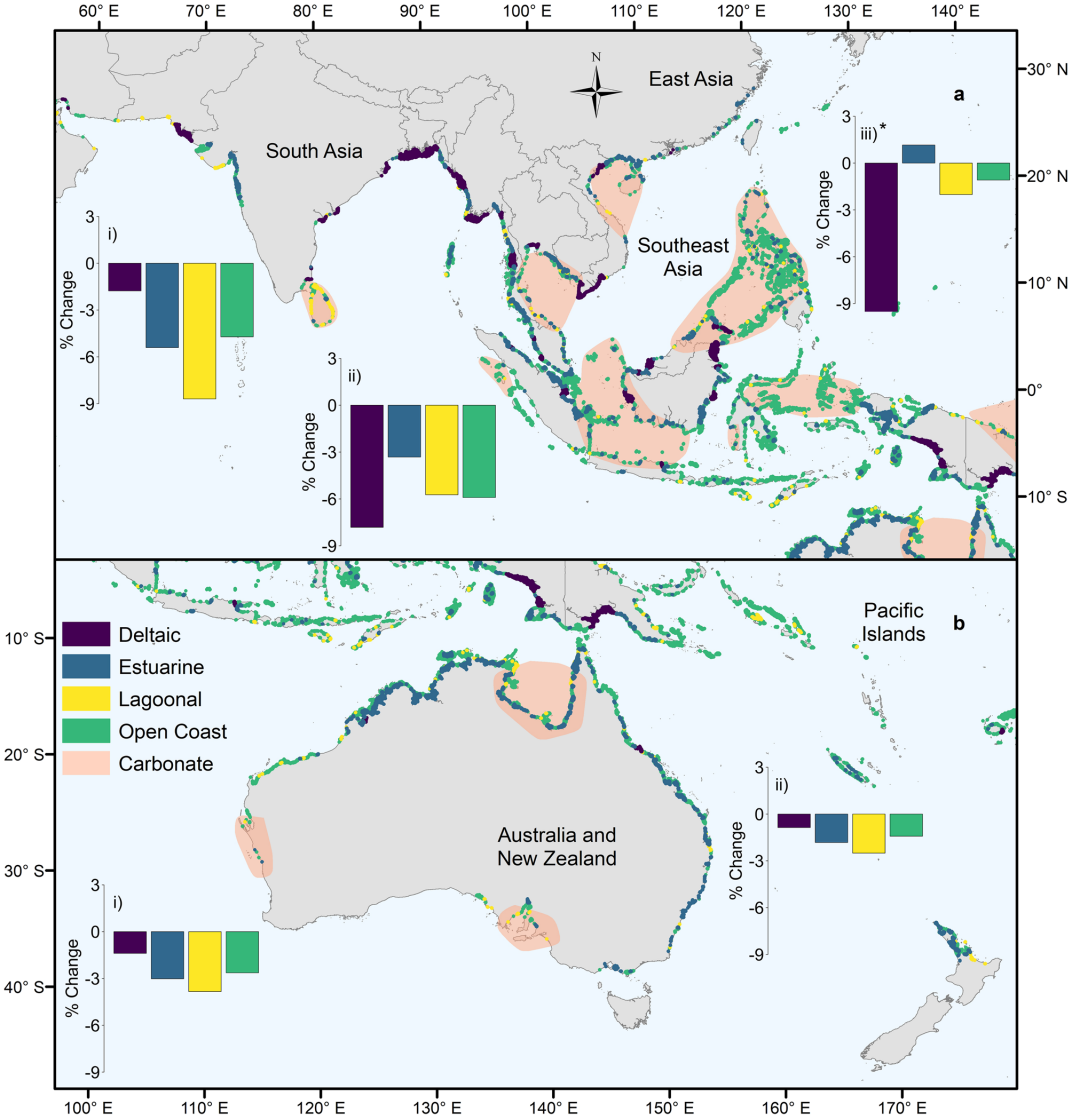
Distribution of deltaic, estuarine, lagoonal and open coast mangrove types, and approximate extent of carbonate sedimentary settings in (ai) the South Asia, (aii) Southeast Asia and (aiii) East Asia regions and (bi) the Australia and New Zealand and (bii) Pacific Islands regions. Bar charts represent the percentage change in area of the different types between 1996 and 2016 at the regional scale. *Value truncated for display, actual value − 33.2%. Adapted from Worthington and Spalding^38^. The map was generated in ArcGIS Desktop version 10.6 software (https://desktop.arcgis.com/en/).

In addition to geomorphic setting, the establishment and stability of mangrove forests are driven by sedimentary processes^20^. Sedimentary setting also determines the density of soil organic carbon stocks^13,15^ and the optimal rehabilitation techniques^20^. We determined the sedimentary setting of mangrove typological patches based on the aquatic inorganic suspended particulate matter concentration and tidal amplitude of the site. Of the 145,595 km^2^ combined GMW 1996, 2007, 2010 and 2016 mangrove extent, 14,657 km^2^ (n = 1,023, 10.1%) was classified as carbonate. In these sediment-poor settings, including isolated oceanic islands in the Caribbean (Fig. 1) and the Pacific (e.g., Solomon Islands, northern Papua New Guinea, Micronesia), the Red Sea (Fig. 2b) and Sri Lanka (Fig. 3a), peat substrate is derived from autochthonous material^29,36^. These habitats appear particularly vulnerable to human disturbance including future sea-level rise^37^.

To examine spatial differences in the proportion of mangroves of different types, the global mangrove distribution was split into ten regions based on those identified in the World Atlas of Mangroves^11^. The proportion of deltaic mangroves was highest in West and Central Africa (56.5%) (Fig. 2a), South America (68.1%) (Fig. 1) and South Asia (82.9%). The role of deltas in preserving the largest remaining intact tracts of mangroves is clear^11^, with deltaic mangroves forming the top 18 largest contiguous mangrove units. Within the biophysical typology, the largest mangrove units are the Niger Delta, Nigeria (6,517 km^2^, Fig. 2a), the deltaic coast of northern Brazil (6,499 km^2^, Fig. 1) and the Sundarbans of India and Bangladesh (6,141 km^2^, Fig. 3a) (Supplementary Table 5). These extensive deltaic mangrove areas form on highly dynamic coastlines that are subject to large inputs of terrigenous material. For instance, mudbanks of the deltaic coast of northern Brazil are rapidly prograding seaward, allowing colonization by mangrove vegetation^39^. Estuarine mangroves formed a large proportion of the mangroves of East Asia (82.0%) (Fig. 3a), Australia and New Zealand (57.9%) (Fig. 3b), and East and Southern Africa (45.6%) (Fig. 2c), with large individual patches in West Africa and Indonesia (Supplementary Table 5). Highly productive river-dominated coastal settings in West Africa and South America are home to some of the largest mangrove trees globally^10^. Conversely, in the xeric areas of the Middle East, there was an absence of estuarine mangroves (Fig. 2b), with mangrove stands characterised by low canopy heights and reduced aboveground biomass^10,40^. Open coast mangroves were more prevalent in Australia and New Zealand (36.6%) (Fig. 3b), the Middle East (69.4%) (Fig. 2b) and the Pacific Islands (42.4%), as well as there being large individual extents in Indonesia (Supplementary Table 5). Lagoonal mangroves are most common in the neotropics^27^ and were largely confined in our typology to North and Central America and the Caribbean region (Fig. 1), but also formed an important component of mangroves in the Middle East (26.9%; Fig. 2b).

### Regional trends in mangrove loss by type

Over the period for which we have data on mangrove extent (1996–2016), we found that, by 2016, the total area of mangrove had been reduced to 135,870 km^2^ (Table 2) from 141,945 km^2^ in 1996. At the global scale, lagoonal mangroves experienced the largest change in area (− 6.9%). Lagoonal areas provide multiple ecosystem services, including tourism and fisheries enhancement^41^; however, degradation of lagoonal environments is often linked to overexploitation of these services^42^.

**Table 2:** Area (km^2^) of mangroves across the regions in 2016 by type.

Region	Deltaic	Estuarine	Lagoonal	Open Coast	Total
Australia and New Zealand	213	5,772	335	3,661	9,982
East and Southern Africa	2,485	3,278	441	1,071	7,275
East Asia	1	130	1	27	158
Middle East	12	0	84	222	318
North and Central America and the Caribbean	1,950	2,663	11,905	4,433	20,951
Pacific Islands	2,598	695	334	2,674	6,302
South America	12,963	3,154	809	2,016	18,942
South Asia	7,041	516	212	645	8,414
Southeast Asia	16,533	13,522	588	13,124	43,767
West and Central Africa	11,176	7,680	285	618	19,760
Atlantic East Pacific	26,089	13,497	12,999	7,068	59,653
Indo West Pacific	28,883	23,914	1,994	21,425	76,217
**Total**	**54,972** (40.5%)	**37,411** (27.5%)	**14,993** (11.0%)	**28,493** (21.0%)	**135,870**

Changes in area for deltaic and open coast mangroves were lower and similar to one another (− 4.3%), while estuarine mangroves experienced the smallest change in area (− 3.1%). Given that delta regions around the world support exceptionally high population densities^43,44^ we expect that historic losses (prior to 1996) in deltaic mangroves through land conversion are likely to have been large. Anthropogenic impacts are also likely to disproportionately impact delta regions into the future^45^, with projected sea-level rise, upstream sediment capture by dams and subsidence increasing vulnerability to flooding^46^.

Our analysis of the sedimentary settings of different mangrove types indicated that losses of carbonate mangroves were more than double (− 8.1%) those of terrigenous areas (− 3.9%). These higher rates of change in carbonate areas were apparent in both lagoonal (− 9.0% versus − 4.9%) and open coast (− 6.9% versus − 3.6%) types. Carbonate mangrove systems may be both more sensitive to natural disturbances such as cyclones, and to anthropogenic threats such as hydrological modification^47^. Disturbances have a longer-term negative impact on carbonate mangroves because they can cause rapid peat collapse and concomitant local increases in relative sea level^48^. Carbonate systems are also potentially more at risk from sea-level rise, as lower suspended sediment concentrations reduce minerogenic contributions to positive elevation change that could match sea-level rise^49^. Rehabilitating organogenic carbonate mangrove systems requires techniques that restore and maintain surface elevation^20^, which are technically challenging (e.g. for marshes^50^) and require monitoring and rapid intervention if restoration trajectories are not being maintained^51^. This analysis provides the first opportunity to identify these at-risk systems, which is important because avoiding peat collapse through mangrove protection is a far more efficient conservation action than attempting to implement technically demanding restoration options.

Over the period 1996 to 2016, the patches that recorded the largest net losses in area (> 100 km^2^, n = 8) were deltaic and a single lagoon (Bahía de Chetumal, northern Belize and southeastern Mexico). Based on changes in the GMW dataset, the units with the largest losses were the Rakhine River Delta, Myanmar (316.2 km^2^); the Mahakam Delta, Kalimantan, Indonesia (277.6 km^2^); the Kayan Delta, Kalimantan, Indonesia (239.8 km^2^); the deltaic coast of northern Brazil (170.1 km^2^), and the Sesayap Delta, Kalimantan, Indonesia (147.4 km^2^). Globally, the drivers of loss in deltaic mangroves vary spatially^52,53^. For instance, expansion of rice agriculture has been highlighted as the major factor in mangrove loss in Myanmar, whilst conversion to aquaculture is more prevalent in Kalimantan, Indonesia^6^ and is also a proximate driver of mangrove deforestation across Latin America and the Caribbean^54^. In addition, shoreline erosion can contribute a significant amount of mangrove loss in deltas^53,55^.

### Quantifying ecosystem structure using the biophysical mangrove typology

**Figure 4:**
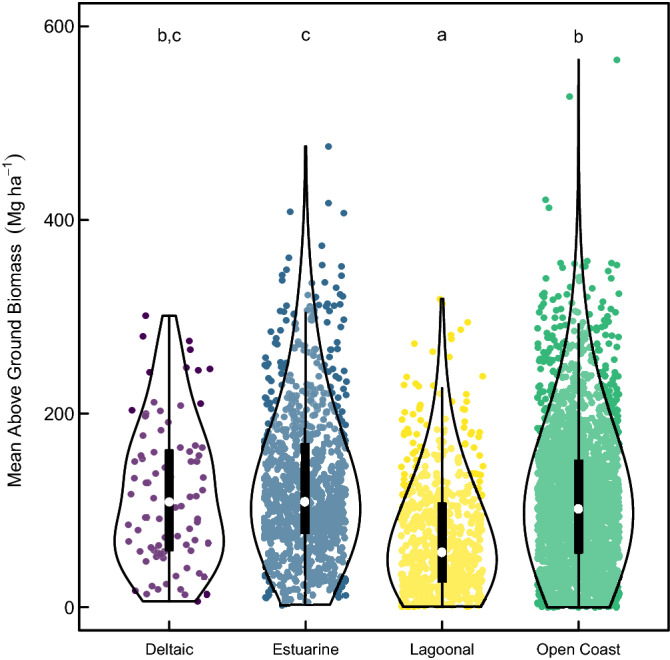
Mean above ground biomass across the four mangrove types. Open circles represent the median value, with box ends representing the upper and lower quartiles and thin lines highest and lowest values excluding outliers (outside 1.5 times the interquartile range above the upper quartile and below the lower quartile). Outline shows data density and spread. Data points shown with a small amount of error added to the x value for display. Letters denote predicted group membership from post-hoc analysis.

The biophysical typology can also contribute to assessing the potential ecosystem structure of an area. Inorganic suspended particulate matter concentration and sediment delivery, aboveground biomass (AGB), tidal amplitude, river dominance, precipitation and substrate composition all influence the structure, species composition and health of mangrove stands and therefore the goods and services they provide. This analysis is the first to attribute global AGB to mangrove-specific types and to investigate the likely role of mangrove type on ecosystem structure. Significant differences between mangrove types were detected (F_3,3771_ = 85.65, *P* < 0.0001); however, the Nagelkerke pseudo-R^2^ = 0.059, suggested low model explanatory power. Post-hoc analysis revealed significant differences (*P* < 0.05) between the AGB in lagoonal, and the AGB in open coast, deltaic and estuarine mangroves, and also between the AGB of estuarine and open coast mangroves. Mean AGB increased from lagoonal (73.5 ± 59.8 S.D. Mg ha^−1^) to open coast (111.5 ± 73.7 S.D. Mg ha^−1^) to deltaic (117.3 ± 73.6 S.D. Mg ha^−1^) to estuarine (126.3 ± 76.3 S.D. Mg ha^−1^) mangroves (Fig. 4). The variation in the data around these averages is high, because typologies span climatic and precipitation gradients, which also influence mangrove biomass^56^. This supports previous plot-scale studies that have shown that estuarine/deltaic mangroves store more biomass and soil carbon than open coast mangroves^16^, and suggests that such patterns exist at multiple scales. This same pattern is not so clearly reflected in mangrove soil carbon, where, under the influence of high minerogenic sediment loads, estuaries and deltas have a much lower percentage of soil carbon per unit volume of soil compared to carbonate or lagoonal settings^13,15^. This is consistent with lower levels of biomass allocation to belowground root material and higher rates of decomposition in deltaic minerogenic settings, which have higher levels of nutrient availability compared to those in carbonate settings^57^.

## Conclusions

### Applications of a global biophysical mangrove typology in ecosystem services and restoration

In this study we extend the utility of the presence/absence time series of mangrove extent by assigning mangroves into discrete types based on their geomorphic and sedimentary setting. The wider landscape context of a mangrove forest is important for identifying the drivers of ecosystem degradation and loss, determining the appropriate restoration technique^20,58^, and assessing the delivery of ecosystem functions and services^12,13,15^. The biophysical typology can also help with projecting the impact of climate change and sea level rise on mangroves^19^, as geomorphic setting determines the boundary conditions affecting mangrove surfaces, and sedimentary settings determines the processes by which mangroves can increase their surface elevations to potentially keep pace with rising seas. This global mangrove biophysical typology therefore has the potential to play a significant role in understanding spatial variability in mangrove threats, ecosystem functions and service values and restoration potential.

## Methods

### Geomorphic setting

We first identified geomorphic features (deltas, estuaries, lagoons, bays) within the mangrove regions of the world using a high resolution coastline, and then determined which mangrove patches were associated with each feature. The first step was therefore to identify coastlines containing either deltas, estuaries, lagoons, bays, or indeed none of these coastal features. Open coast mangroves are areas associated with bays, or no coastal embayment. The other mangrove types were associated with their respective coastal feature. Deltas, estuaries, lagoons and bays are generally all characterised by rapid changes in direction of the mapped coastline and thus we created a GIS dataset (ArcGIS Desktop version 10.6, https://desktop.arcgis.com/en/) of coastal embayment polygons (CEPs) for the mangrove regions of the world. This initial dataset was based on the Global Administrative Boundaries layer (https://www.gadm.org/), amended by a small number of patches from the World Vector Shoreline (https://shoreline.noaa.gov/data/datasheets/wvs.html), where the latter had greater definition of the coastline. CEPs were created by preparing the coastline vector before running a Euclidean Allocation and Euclidean Distance analysis on the boundaries to identify individual potential bays, lagoons, deltas and estuaries; essentially indents in the coastline. The resulting dataset consisted of 12,301 CEPs. CEPs were selected on landmasses greater than 30 km^2^ and within 20 km of the union of the GMW 1996, 2007, 2010 and 2016 maps, a high-resolution global dataset of mangrove distribution (further details given in Supplementary Section 2.1).

#### Classifying coastal embayment polygons

Delta CEPs were identified using two procedures. Firstly, deltas (n = 81) in mangrove areas were identified from the World Atlas of Mangroves^11^, The Major River Deltas Of The World^59^ and Major World Deltas: A Perspective From Space^60^. Secondly, CEPs were assessed based on the number of drainage outlets to the ocean. Those with more than two outlets were identified and visually assessed. CEPs were classified as deltas based on polygon shape, having a large catchment area with multiple river flowlines (distributaries), and an internet search identifying reference to the river having a delta (n = 21). Delta extents were created using either those already derived in the Deltas at Risk dataset https://www.globaldeltarisk.net/data.html or manually using online sources and Google Earth (Google Earth Pro version 7.3.3.7699, https://www.google.com/earth/). The delta extents were used to combine multiple CEPs into a single unit (further details given in Supplementary Section 2.2).

Delta CEPs and CEPs identified visually as errors were removed before a random forest classifier was used to assign the remaining CEPs into three types (‘bays’, ‘estuaries’ and ‘lagoons’). The random forest classification was based on ten variables describing the shape of the polygons, their associated upstream hydrological catchment and the amount of precipitation entering the catchment (Supplementary Table 1). The hydrological catchment data were accessed from the (HydroSHEDS) dataset (https://www.hydrosheds.org/). We identified HydroSHEDS river network flowlines that intersected with the CEPs, and the HydroSHEDS watershed polygons that intersected with these selected flowlines were selected and aggregated to form a single catchment extent (further details given in Supplementary Section 2.3). The amount of precipitation moving through the river network to each CEP was of the form of monthly precipitation and accessed from https://www.earthenv.org/streams. The precipitation data was developed to fit alongside the HydroSHEDS framework^61^ (further details given in Supplementary Section 2.3).

The random forest (randomForest package^62^) analysis using 100,000 trees was initially run on a CEP training dataset containing 800 bays, 71 lagoons and 300 estuaries (total n = 1,171) in R (version 3.4.4^63^), with 20% of the data randomly selected for model validation. All other parameters were left as the default. Selection of the CEPs for the training dataset was undertaken by expert annotation and was not randomised. Instead 100 bays in each of the following mangrove regions were included: North America, South America, West Africa, Southeast Africa, Middle East, Asia, Australasia, the Pacific. Estuary and lagoon CEPs were visually identified using a global typology of nearshore coastal systems^64^, from ‘tidal systems’ or ‘lagoon’ coastal types respectively.

The resulting random forest model was fitted to the remaining CEP dataset. A random sample of 500 bays and all estuary and lagoon CEPs (n = 1,271) were visually inspected at a 1:500,000 scale in ArcGIS (ArcGIS Desktop version 10.6, https://desktop.arcgis.com/en/) to assess the accuracy of the model and correct misclassifications. Visual assessment was based on the size and shape of each CEP, the river catchment inputs to each feature and the wider geographical context (further details given in Supplementary Section 2.4).

Given misclassifications from the initial random forest model, the process was repeated with a further 75,000 trees on the non-visually assessed bay CEPs using the original 1,171 training points and the visually inspected and corrected CEPs from the first random forest model. The second random forest iteration was then fitted onto these remaining bay CEPs. If there was a disagreement in the predicted type between the two random forest models, the CEP was visually assessed and, where necessary, corrected (results of the Random Forest given in Supplementary Section 2.5 and limitations of the methodology in Supplementary Section 4).

#### Attributing mangroves to the biophysical typology

We then determined which mangrove patches were associated with the classified CEP. The mangrove extent used as a framework for the biophysical typology was the union of the GMW 1996, 2007, 2010 and 2016 maps, with mangrove patches classified into one of four types: deltaic, lagoonal, estuarine or open coast. Assigning the mangrove patches to a type and an individual CEP followed a stepwise procedure (see Supplementary Figs. 2, 3). While there were many steps, they can be broadly classified into three aims: firstly; ensuring that all existing mangrove patches could be assigned to a single CEP by splitting very large mangrove patches where appropriate or those mangrove patches intersecting two or more CEPs; secondly allocating patches that directly intersected to a single CEP and finally; assigning mangrove patches that did not directly intersect with a CEP to the appropriate typological unit using HydroSHEDS catchment boundaries and distance between CEPs and mangrove patches. Following the stepwise procedure, several rounds of visual quality assessment and corrections were carried out (further details given in Supplementary Section 2.6).

### Sedimentary setting

For the non-deltaic and estuarine patches we further sought to determine the sedimentary setting. Following Balke and Friess^20^ we determined the sedimentary setting of the mangrove typological patches based on the aquatic inorganic suspended particulate matter concentration and tidal amplitude of the site. Two hundred and forty monthly inorganic suspended particulate matter concentration (g/m^3^) global data rasters were downloaded from the Globcolor website https://www.globcolour.info and the mean inorganic suspended particulate matter concentration for each pixel was calculated. A tidal data raster from the Finite Element Solution tide model, FES2014, was downloaded from AVISO + products (https://www.aviso.altimetry.fr) (further details on the data sources given in Supplementary Section 3).

Training points were taken from 152 locations with known typological (riverine or non-riverine) and sedimentary (terrigenous or carbonate) status and within 10 km of the GMW maximum extent. These were identified through reference to the literature or the authors’ own diverse field experiences. The tidal amplitude value and mean inorganic suspended particulate matter concentration nearest to the training location was determined in ArcGIS (ArcGIS Desktop version 10.6, https://desktop.arcgis.com/en/) and then imported into R (version 3.6.2^63^).

Estuarine or deltaic sites (n = 70) were removed from the data set and a two-sided binomial generalized linear model with a logit link was fitted to the remaining 82 sites in R (version 3.6.2^63^), with the resulting model being used to classify the lagoonal and open coast mangroves as either carbonate or terrigenous. The pseudo-R^2^ was calculated as the (null deviance – residual deviance)/ null deviance and was 46.8%. M_2_ tidal amplitude was a significant predictor (z = 4.5, *P* =  < 0.001); however mean inorganic suspended particulate matter concentration was a non-significant predictor (z = 0.95, *P* = 0.34), but was retained in the model. The model misclassified six (of n = 29) carbonate and nine (of n = 53) terrigenous sites. The resulting model was then mapped in ArcGIS to determine whether lagoonal and open coast mangrove patches were in a terrigenous or carbonate setting. Estuarine and deltaic mangroves were universally classed as terrigenous.

### Mangrove above ground biomass

Global data on the AGB of mangroves (Mg ha^−1^) was downloaded from (https://daac.ornl.gov/)^65^. The AGB values were derived from remotely sensed data on basal area weighted height combined with field measurements^10^. The AGB values were developed for the year 2000 using the Giri and colleagues^23^ global mangrove distribution dataset. This resulted in a mismatch with our biophysical mangrove typology, which was based on the combined area of the GMW 1996, 2007, 2010 and 2016 timesteps. Therefore, the AGB raster dataset was converted to points that were then spatially joined to our biophysical typology dataset and the mean AGB value in each typological unit calculated (deltaic type n = 84, estuarine type n = 907, lagoonal type = 591, and open coast type = 2,193).

To determine whether there was a significant difference in the AGB between the mangrove types, a two-sided generalized least squares model was developed using the ‘nlme’ package in R^66^. Validation of the initial model, undertaken by creating histograms of the normalized residuals and plotting the normalized residuals against the fitted values and the covariate^67,68^, suggested issues with non-normality and heteroscedasticity. Therefore, the square root of the mean AGB was used and a variance structure for mangrove type was included^69^, with a clear improvement in residual validation plots. Post-hoc tests on the difference between the estimated marginal means of each mangrove type were computed using the ‘emmeans’^70^. The Nagelkerke pseudo-R^2^ was calculated using ‘rcompanion’^71^.

## Supplementary information

Supplementary information

## Data Availability

The global biophysical mangrove typology is available for download from the Ocean Data Viewer (https://data.unep-wcmc.org/).
