# Supplementary material for: A global biophysical typology of mangroves and its relevance for ecosystem structure and deforestation
Source: Sci Rep. 2020 Sep 4;10:14652. doi: 10.1038/s41598-020-71194-5 (PMC7473852; doi:10.1038/s41598-020-71194-5)
Supplement: Supplementary file 1 — Supplementary information [file 41598_2020_71194_MOESM1_ESM.pdf]

Supplementary Information

A global biophysical typology of mangroves and its relevance for ecosystem structure and deforestation

Thomas A. Worthington[1]§*, Philine S.E. zu Ermgassen[2]§, Daniel A. Friess[3], Ken W. Krauss[4], Catherine E. Lovelock[5], Julia Thorley[6], Rick Tingey[7], Colin D. Woodroffe[8], Pete Bunting[9], Nicole Cormier[10], David Lagomasino[11, 12], Richard Lucas[9], Nicholas J. Murray[13], William J. Sutherland[1], Mark Spalding[1, 14].

§Joint first authors, * Correspondence to taw52@cam.ac.uk

1. Conservation Science Group, Department of Zoology, University of Cambridge, Cambridge CB2 3QZ UK

2. Global Change Group, School of Geosciences, Grant Institute, Kings Buildings, University of Edinburgh, Edinburgh, EH9 3FE UK

3. Department of Geography, National University of Singapore, 1 Arts Link, Singapore, 117570 Singapore

4. U.S. Geological Survey, Wetland and Aquatic Research Center, 700 Cajundome Blvd, Lafayette, LA, 70506 USA

5. School of Biological Sciences, University of Queensland, St. Lucia, QLD, 4072 Australia

6. Independent GIS Consultant, Penzance, UK

7. Spatial Support Systems, LLC, Cottonwood Heights, UT, 84121 USA

8. School of Earth Atmospheric and Life Sciences, University of Wollongong, Wollongong, NSW, 2522 Australia

9. Department of Geography and Earth Sciences, Aberystwyth University, Aberystwyth, Wales, United Kingdom

10. Department of Earth and Environmental Sciences, Macquarie University, Level 4, 12 Wally's Walk, Sydney, NSW, 2109 Australia

11. Department of Coastal Studies, East Carolina University, Wanchese, NC, 27981 USA

12. Biospheric Sciences Laboratory, NASA Goddard Space Flight Center, Greenbelt, MD, 20771 USA

13. College of Science and Engineering, James Cook University, Townsville, QLD, 4811 Australia.

14. The Nature Conservancy, c/o Department of Physical, Earth, and Environmental Sciences, University of Siena, Pian dei Mantellini, 53100 Siena, Italy

1. Developing a Typology

1.1 Deltas and Deltaic Mangroves

A delta is a discrete shoreline sedimentary protuberance formed where a river enters an ocean, semi-enclosed sea, estuary, lake, or lagoon and supplies sediment more rapidly than it can be redistributed by basinal processes[1]. In this typology, deltas refer to those occurring in open coastal settings rather than smaller deltas forming within other embayments (lagoons, bays or estuaries). While deltas take a range of forms depending on the relative dominance of riverine, tidal and wave processes[2] they are often typified by a wide fan-shaped plain, typically traversed by both active and abandoned distributary channels. Deltas in this typology are synonymous with the river-dominated type of Thom[3] and Woodroffe[4], where large volumes of terrigenous sediment are transported by rivers, leading to rapid deposition and progradation.

1.2 Estuaries and Estuarine Mangroves

An estuary is a 'semi-enclosed coastal body of water with a free connection to the open sea and within which seawater is measurably diluted with fresh water derived from land drainage'[5]. Estuaries can differ significantly in their geomorphology, tidal influence, riverine and sediment inputs and salinity[6]. Estuaries in this typology align with aspects of the tide-dominated classes of Thom[3] and Woodroffe[4] and the estuary types of Twilley and Rivera-Monroy[7] and Woodroffe and colleagues[8]. These coastal features are characterised by funnel shaped main channels and bidirectional tidal flows[3] and to capture this latter point, and to ensure that we excluded non-riverine embayments, we trained our model towards coastal embayment polygons (CEPs) linked to large catchment areas and high precipitation inputs. These estuarine features have sediments with a significant clastic component from a range of freshwater and marine sources[7]. Thom[3] and

Woodroffe[4] highlight these features being present in macrotidal regions; given our use of feature shape as a driver for classifying CEPs, CEPs from micro and mesotidal areas are also classified as estuaries in our typology. While tidal forcing is defined as a dominant driver by Thom[3] and Woodroffe[4], it was not directly incorporated into our model. In line with Thom's[3] allochthonous coast setting, estuarine mangroves in our typology were all assigned a terrigenous sedimentary type.

1.3 Lagoons and Lagoonal Mangroves

Coastal lagoons have been defined as 'a shallow coastal water body separated from the ocean by a barrier, connected at least intermittently to the ocean by one or more restricted inlets, and usually oriented shore-parallel'[9]. Many are naturally ephemeral with water exchange impacted by shifting sediments closing or opening marine connections[10]. Our lagoon patches would be equivalent to carbonate sand/shingle barrier patches or the terrigenous wave dominated barrier lagoons in Thom[3] mangrove typology. The terrigenous lagoons are found in areas of high wave energy and low freshwater inputs, whereas in carbonate setting lagoons on low-energy coasts form behind barrier islands, spits or bay barriers[3], with mangroves establishing in these sheltered settings[4,8].

1.4 Bays and Open Coast Mangroves

Embayments are less complex than the other three coastal features and can take the form of 'rounded bays, highly indented bays with convolute shorelines, or narrow and tapered drowned river valley systems'[11]. Within our typology open coast mangroves would incorporate several of the divisions used in other typologies (Table 1). In terrigenous settings they would be analogous

to drowned bedrock valleys[3], within which mangroves can form in the sheltered environments[4]. In carbonate areas open coast mangroves form on oceanic islands, and behind coral reefs and carbonate banks[4,7]. These areas are often typified by a microtidal regime, with sediment formed of mangrove based peat rather than from terrigenous inputs[4,8].

2. Geomorphic Setting

2.1 Analysis Extent

Giri and colleagues[12] global mangrove distribution dataset was used to set the extent of the analysis and select the CEPs for the random forest classification. Mangroves patches were first converted to convex hull polygons and then buffered 20 km. However, with the release of the updated Global Mangrove Watch (GMW, version 2, released 2019) additional CEPs required classification. A 20 km buffer was created around the union of the GMW 1996, 2007, 2010 and 2016 timesteps. CEPs that were in the GMW 20 km buffer but had not been assigned to a type previously were identified. These new CEPs were visually inspected, assigned as either a delta, lagoon, estuary or bay following the same procedure below, and merged with the previously defined CEPs. All spatial data manipulation in this, and subsequent, sections undertaken in ArcGIS (ArcGIS Desktop version 10.6, https://desktop.arcgis.com/en/), unless otherwise stated.

2.2 Classifying Delta Polygons

Delta CEPs were identified using two procedures. Firstly, we identified deltas (n = 81) in mangrove areas from those listed in the World Atlas of Mangroves[13], The Major River Deltas of the World[14] and Major World Deltas: A Perspective From Space[15]. Outlines for these deltas were created using either those already derived in the Deltas at Risk dataset http://www.globaldeltarisk.net/data.html or manually using online sources and Google Earth Pro (Google Earth Pro version 7.3.3.7699, https://www.google.com/earth/). When creating the delta outline polygon, multiple neighbouring CEPs were included based on an examination of their connectivity in Google Earth Pro. The aim of the delta outline polygon was to incorporate the correct CEPs rather than get the overall delta shape especially accurate.

Secondly, CEPs were assessed based on the number of outlets to the ocean. Outlets were identified by creating an ocean polygon for mangrove areas. The ocean polygon was the inverse of a combined extent of landmasses that had mangroves on them as well as the CEPs. Line segments over 200 m from the CEPs that intersected the ocean polygon were determined to be the CEPs' ocean outlet. This procedure identified several CEPs with no outlets. A small number were the result of the 200 m threshold described above and were manually corrected. The remaining were CEPs that were contiguous with another CEP and were therefore merged. Once the outlets had been created, CEPs with more than two outlets were identified and visually assessed. CEPs were classified as deltas based on polygon shape, having a large catchment area with multiple river flowlines, and an internet search identifying reference to the river having a delta (n = 21). As before an extent outline for the delta was created.

2.3 Hydrological Catchments and Precipitation

To determine the area of the associated upstream hydrological catchment and the amount of precipitation associated with each CEP, we assigned catchments to each of the CEPs. We did so based on the watershed boundaries and stream networks from the 'hydrological data and maps based on shuttle elevation derivatives at multiple scales' (HydroSHEDS) dataset (http://www.hydrosheds.org/). The HydroSHEDS data are primarily derived from the Shuttle Radar Topography Mission 3 arc-second (~90m at the equator) digital elevation model[16]. The watershed boundaries and stream networks used to create the upstream hydrological catchments were at a resolution of 15 arc-second (~500m at the equator). We identified HydroSHEDS river network flowlines that intersected with the CEPs and then those HydroSHEDS watershed

polygons that intersected with these selected flowlines were selected and aggregated to form a single extent. Catchment area was calculated as the sum of the aggregated extent (in km^2). These catchments were also used as a basis with which to calculate the precipitation reaching each CEP. The data was of the form of monthly precipitation and accessed from http://www.earthenv.org/streams, which is based on WorldClim climate data at a 1 km resolution for the period 1950 – 2000[17]. The climate data is overlaid on the HydroSHEDS 30 arc-second (~1km at the equator) watersheds and provides an estimate of the sum of precipitation transitioning through the river network[17]. The data containing the monthly values were imported into R (version 3.4.4[18]) and a raster layer representing the annual precipitation sum was created. The maximum precipitation in each sub-catchment was identified and the total value for each CEP upstream hydrological catchment calculated. Given the difference in spatial resolution of the catchment and precipitation datasets (15 arc-seconds vs. 30 arc-seconds, respectively) there was a minor spatial mismatch between the catchment polygon boundaries and the precipitation raster layer. This generally resulted in the very downstream pixels of the precipitation layer being outside the catchment boundary and therefore not counting to the precipitation result.

Supplementary Table 1: Variables used in the random forest model to discriminate between estuary, lagoon and bay coastal embayment polygons (CEPs). The map insets were generated in ArcGIS Desktop version 10.6 software (https://desktop.arcgis.com/en/).

**Variable**	**Description**	**Hypothesis**	**Example**
Polygon Area	The area of the coastal embayment polygon (km^2)	Bays in general would be smaller geomorphological features	
Polygon Perimeter	The perimeter of the coastal embayment polygon (m)		
Ocean Outlets	The number of outlets to the ocean for each coastal embayment polygon		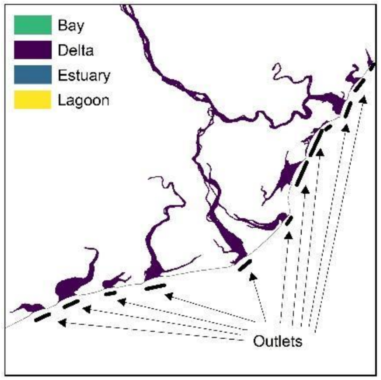
Length of Ocean Outlets	The total length of the ocean outlets (m)	Lagoons will have short lengths of ocean outlets	

Length Ratio	The ratio of the maximum Euclidean distance between the ends of each coastal embayment polygon's outlet(s) and the maximum side length of a minimum bounding rectangle encompassing the coastal embayment polygon	The ratio will be close to one for bays but larger for estuaries and larger still for lagoons	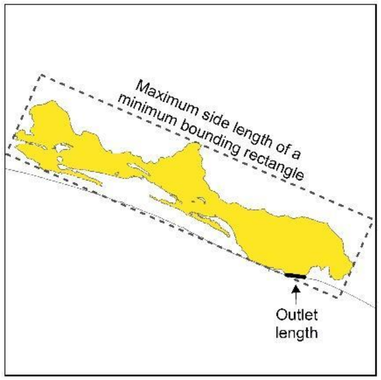
Angle Difference	The difference in angle between the line representing the maximum Euclidean distance between the ends of each coastal embayment polygon's outlet(s) and the line representing maximum side length of a minimum bounding rectangle encompassing the coastal embayment polygon	Bays and lagoon are usually parallel to the prevailing direction of the coastline, whereas estuaries are usually perpendicular and therefore have different angle attributes.	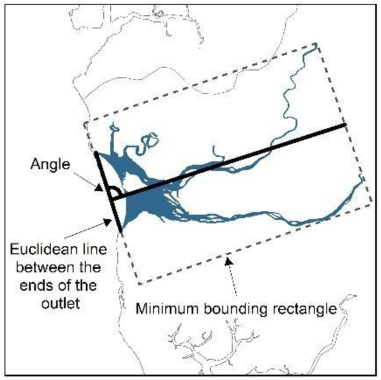

Convex Ratio	The ratio of the area of the coastal embayment polygon and the area of minimum bounding convex hull encompassing the coastal embayment polygon	Owing to the simple crescent shape the ratio will be close to one for bays and higher for the estuaries and lagoons.	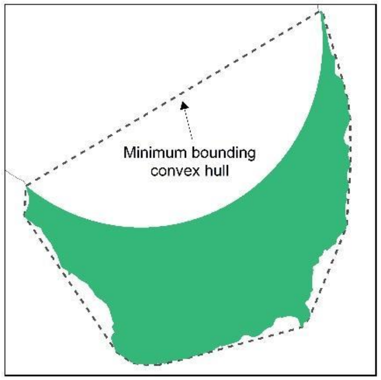
Mouth Perimeter Ratio	The ratio of the total length of the mouths to the perimeter of the coastal embayment polygon	The ratio will be very high for lagoons due to the small outlet versus the large size	
Catchment Area	The area (km^2) of the upstream hydrological catchment derived from HydroSHEDS data	The catchment area will be greatest for estuaries, followed by lagoons, with small catchment areas around bays.	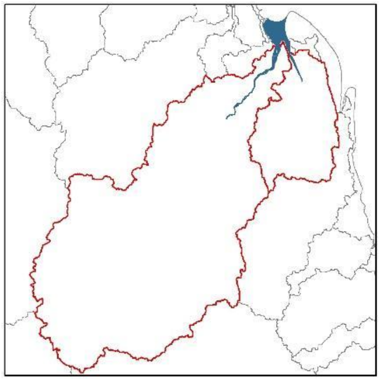

Maximum Precipitation	The sum of the maximum annual precipitation entering each coastal embayment polygon's upstream hydrological catchment	More precipitation will enter the catchments of estuaries than lagoons or bays	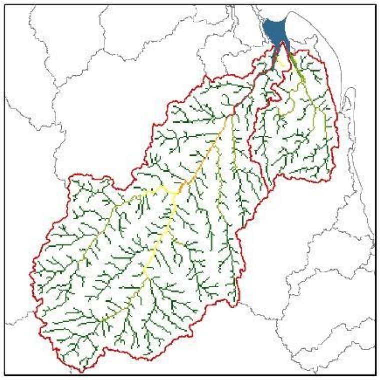

2.4 Random Forest Accuracy Assessment

To examine the accuracy of the predictions from the random forest model, a random sample of 500 bays was selected and the prediction accuracy visually assessed at a 1:500,000 scale in ArcGIS (ArcGIS Desktop version 10.6, https://desktop.arcgis.com/en/). Given the difficulty in classifying lagoons, with many predicted to be estuaries, all CEPs (n = 1271) classified as lagoons and estuaries were visually inspected at a 1:500,000 scale. Visual classification was based on the size, shape of each CEP and river catchment inputs to each feature. The wider geographical context of the CEP was also used; for example, unless there was an obvious large river system, CEPs in deserts and xeric shrublands areas (e.g., the Middle East, parts of Africa based on the Terrestrial Ecoregions of the World[19]) would not be classified as estuaries. Likewise, if a CEP was on a stretch of coast containing many other estuary CEPs, it would be more likely to be classified as such. Where necessary to aid classification, the feature was viewed using high resolution aerial imagery in Google Earth Pro (version 7.3.1.4507, 2018, Google Inc.). Indicative features visible in Google Earth Pro included sediment from the catchment, indicating significant freshwater input in the case of estuaries, while lagoons were typically identified by shallow water and the presence of barrier islands (e.g., Texas, U.S.A.). Bays could be a range of sizes, would have little surrounding catchment inputs and would be characterised by deep water.

2.5 Random Forest Results

The random forest model was driven by the variables; convex ratio, catchment area, maximum precipitation, angle difference, length ratio and mouth perimeter ratio (Supplementary Fig. 1). When tested on the validation data, the model was accurate at classifying bays (class error, 0.6%)

and estuaries (8.3%), but not lagoons (57.1%, Supplementary Table 2). From the remaining 7563 CEPs, the model classified 6292 bays, 965 estuaries and 306 lagoons. The visual accuracy assessment found that 99% of the 500 randomly selected bays had been correctly classified, whereas this reduced to 71.6% for estuaries and 49.3% for lagoons (Supplementary Table 3). The second random forest iteration was driven by the same variables. Type prediction errors for the validation data were bays 7.3%, estuaries 7.8% and lagoons 45.7% (Supplementary Table 4), with decrease in the accuracy in bays somewhat offset by improvements in prediction of estuaries and especially lagoons. When the second random forest iteration was transferred onto the remaining 5792 bay CEPs there was a high level (98.6%) of agreement with only 81 CEPs (estuary = 76, lagoon = 5) being classified as a different type.

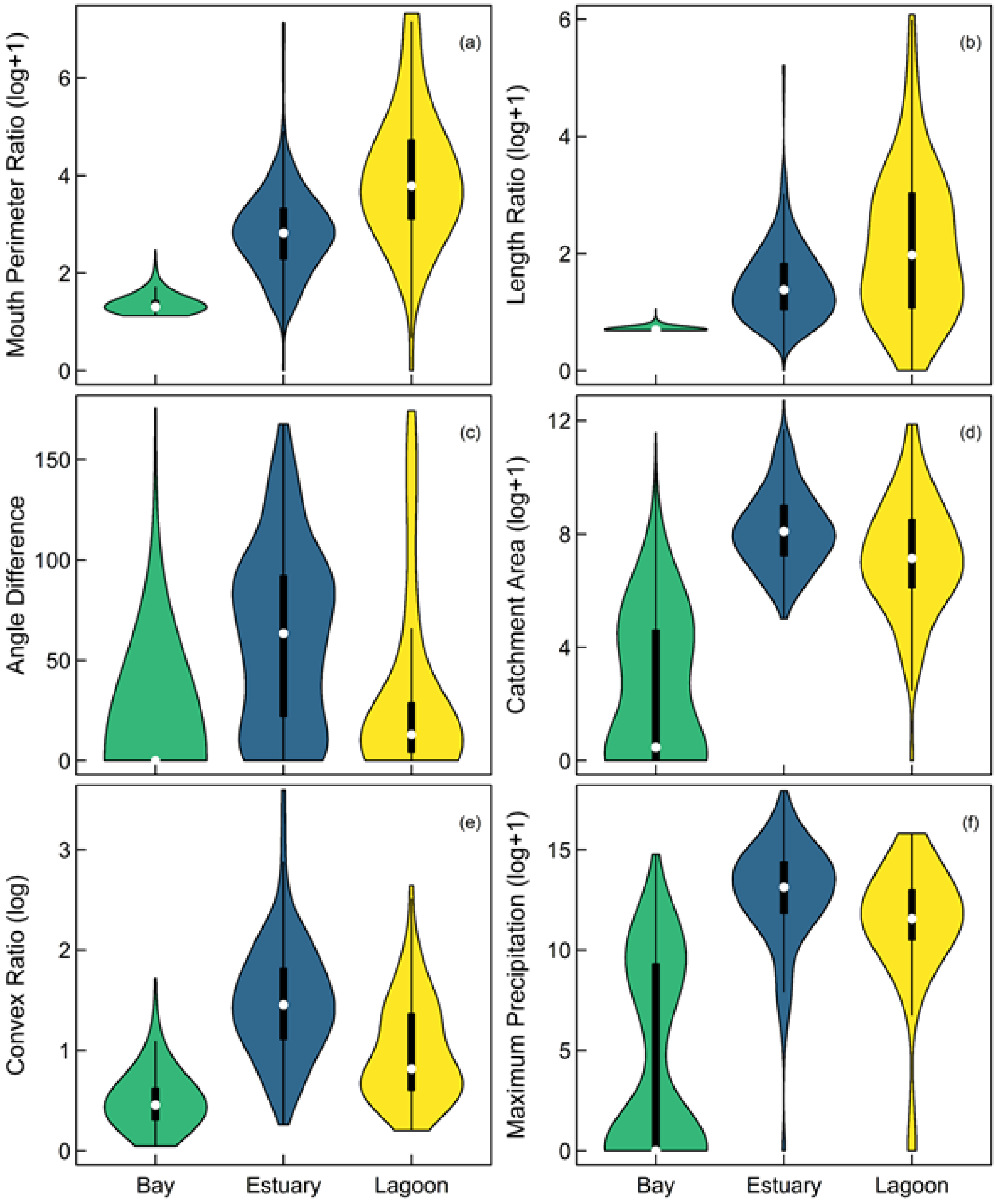

Supplementary Figure 1: The distribution of values for the six most important variables in the random forest across the types. The open circle represents the median value, with box ends representing the upper and lower quartiles and thin lines highest and lowest values excluding outliers. Shaded areas show data density and spread.

Supplementary Table 2: Confusion matrix showing the predicted versus the observed classifications of the random forest model predicted onto the validation data.

**Observed**	**Predicted**			
	Bay	Estuary	Lagoon	**Accuracy**
Bay	159	1	0	**99.4%**
Estuary	5	55	0	**91.7%**
Lagoon	1	7	6	**42.9%**
**Total**	**165**	**63**	**6**	**94.0%**

Supplementary Table 3: Confusion matrix showing the predicted classification of estuaries and lagoons of the random forest model versus the observed classifications from ArcGIS.

**Observed**	**Predicted**			
	Bay	Estuary	Lagoon	**Accuracy**
Bay	0	0	0	**-**
Estuary	144	691	130	**71.6%**
Lagoon	130	25	151	**49.3%**
**Total**	**274**	**716**	**281**	**66.2%**

Supplementary Table 4: Confusion matrix showing the predicted versus the observed classifications of the second random forest model predicted onto the validation data.

**Observed**	**Predicted**			
	Bay	Estuary	Lagoon	**Accuracy**
Bay	290	18	5	**92.7%**
Estuary	11	188	5	**92.2%**
Lagoon	4	28	38	**54.3%**
**Total**	**305**	**234**	**48**	**87.9%**

2.6 Attributing Mangroves to Typology

Assigning the mangrove patches to a type and an individual CEP followed a stepwise procedure (Supplementary Fig. 2 & 3). Firstly, as large mangrove patches were likely to be near multiple types or multiple CEPs, mangrove patches greater than 5000 hectares (50 million m^2) were identified and visually inspected in ArcGIS (ArcGIS Desktop version 10.6, https://desktop.arcgis.com/en/). Where appropriate the mangrove patches were split between CEPs, and/or large extents of open coast mangrove classified. Mangrove patches that intersected with the CEPs (estuaries, lagoons, and deltas only) were identified and the number of intersections recorded. Mangroves patches (n = 193) that intersected with multiple CEPs (estuaries, lagoons, and deltas only) were visually assessed and the patches were split based on approximate HydroSHEDS catchment boundaries, minimising the number of patches created and the geographic structure of the mangrove patches. Patches were assigned to the correct CEP, or to open coast mangroves. Following the three rounds of splits, the final dataset was 739,965 mangrove patches.

Mangrove patches that were on landmasses that had no estuary, lagoon or delta CEPs were classified as open coast. Mangrove patches that intersected with a single (following previous splits) estuary, lagoon or delta CEP were assigned to that CEP type. Mangrove patches that intersected with a bay (or multiple bay) CEPs were assigned as open coast.

The HydroSHEDS dataset was also used to designate mangrove patches as estuarine, deltaic or lagoonal. In this case the upstream hydrological catchment to each CEP was created by identifying the HydroSHEDS river network flowlines that intersected with the estuary, delta or

lagoon CEP. The flowlines were given the ID of the intersecting CEP, and those intersecting multiple CEPs were visually assessed and manually assigned. Similarly, HydroSHEDS watershed polygons that intersected with an estuary, delta or lagoon CEP were identified and assigned the ID of that CEP. As before those intersecting multiple CEPs were manually assigned. In addition, HydroSHEDS watershed polygons that intersected with the flowlines previously identified were assigned to the corresponding coastal embayment. The selected HydroSHEDS basins were merged based on the coastal embayment ID to form a single catchment extent.

The boundary vertices of the second version of the hydrological catchments that intersected with the estuary, lagoon or delta CEPs were buffered by 3000 m. The buffer was used to identify mangrove patches that were clearly within the catchment of a single CEP, as mangrove patches intersecting the buffer could potentially be assigned to multiple CEPs. Patches that did not intersect with the buffer but did intersect with a catchment were assigned to the CEP linked to the catchment. Given their large extent, deltas were visually checked and manual corrections to include mangrove patches falling outside the catchment boundary or deltaic islands were made. Given a lack of complete coverage in the catchment extents, a small number of mangrove patches that were closer to a CEP than the ocean were identified, visually assessed and assigned. The distance between mangrove patches that did intersect with the 3000 m catchment buffer and the CEP was calculated and those that were <= 500 m from an estuary, lagoon or delta CEP were assigned to it, followed by those that were <= 500 m from a bay CEP and therefore classed as open coast.

The distance between the remaining unassigned mangrove patches, the vertices of the previously assigned mangrove patches and CEPs was calculated (maximum distance 10,000 m). If a patch was >10,000 m from an estuary/delta/lagoon CEP it was assigned as open coast. Mangrove patches that were within 1000 m of an estuary/delta/lagoon CEP or a mangrove patches already assigned as estuarine/deltaic/lagoonal were assigned to that feature. If a patch had not already been assigned as estuarine/deltaic/lagoonal and was < 1000 m from a bay or an already assigned open coast mangrove patch it was assigned as open coast.

The remaining mangrove patches were classified using an iterative process whereby those mangrove patches <= 1000 m from an already assigned estuarine/deltaic/lagoonal mangrove patches were assigned to the nearest patch. The result was then merged with the already assigned mangrove patches and repeated for distances of 1000 m, 500 m, 250 m and 100 m. Remaining mangrove patches were assigned as open coast. Whereas, estuarine/deltaic/lagoonal mangrove patches had been assigned to the corresponding CEP to form a distinct unit, open coast mangroves were in individual patches. Therefore, to create larger open coast typological units, open coast mangrove patches were aggregated based on a distance of 5000 m.

Following the stepwise procedure several rounds of visual quality assessment and corrections were conducted. Firstly, several bay CEPs in the Florida Everglades and Mexico were reassessed and were determined to be part of larger lagoonal complexes and therefore reclassified as lagoons and the mangrove patches reassigned. In addition, several CEPs were part of a larger deltas or estuaries, these were merged, and the mangrove patches reassigned. Secondly, each estuary/delta/lagoon CEP was visually assessed, and misclassified mangrove patches were

reassigned. This generally related to inland mangrove patches classified as open coast rather than estuarine/deltaic/lagoonal or coastal patches that were converted to open coast if they were beyond the probable influence of the coastal embayment feature. Several co-authors provided comments on areas that had been misclassified which were assessed and changed where necessary, including the reclassification of a number of delta CEPs to estuaries.

A quality check was carried out on large aggregations of open coast mangrove. Patches of open coast mangrove within 1000 m of each other were identified and those aggregated patches > 100 hectares were visually assessed in ArcGIS (ArcGIS Desktop version 10.6, https://desktop.arcgis.com/en/). This visual assessment addressed the following issues. Firstly, incorrect assignment of open coast mangrove patches to lagoonal, based on weaknesses in the original coastline layer which did not capture inland water bodies. Lagoons were identified using photographic imagery in ArcGIS (https://www.arcgis.com/home/item.html?id=10df2279f9684e4a9f6a7f08febac2a9) and large numbers of open coast mangrove patches were converted along the high energy coasts and areas of considerable longshore sediment transport. Secondly, conversion of lagoons to open coast where channels between islands were misclassified as coastal embayments. Thirdly, Northern Cuba, the complex of mangroves and near continuous islands along the Sabana Archipelago and the Camagüey Archipelago were poorly captured by the original embayment mapping. These are highly protected and largely enclosed mangroves and were therefore entirely classified as lagoonal. Fourthly, Florida, considerable manual interventions due to complexities and mis-classifications of coastline. The manual interventions following the stepwise procedure (N.B. there had be minor manual intervention during the stepwise procedure) resulted in variation in

the number of patches assigned to each type particularly increasing the amount of lagoonal mangrove: estuarine (+0.2%), deltaic (-1.9%), lagoonal (+24.4%) and open coast (-5.6%).

A final quality check was undertaken to reduce the number of very small typological units. All units <1km^2 were visually assessed and errors (small numbers of misclassified pixels) were corrected. In addition, small open coast units were either aggregated together or joined to larger open coast units. This was achieved by splitting the open coast units into large (>1 km^2) and small (<1 km^2) classes and merging into the large units those small units that were within 10,000 m. The new aggregations were again split into large and small classes and a further merge of small units within 10,000 m of a large unit was undertaken. In total this process was repeated six times until there were no small units were within 10,000 m of a large unit. Finally, the remaining small units were aggregated together based on a distance of 10,000 m.

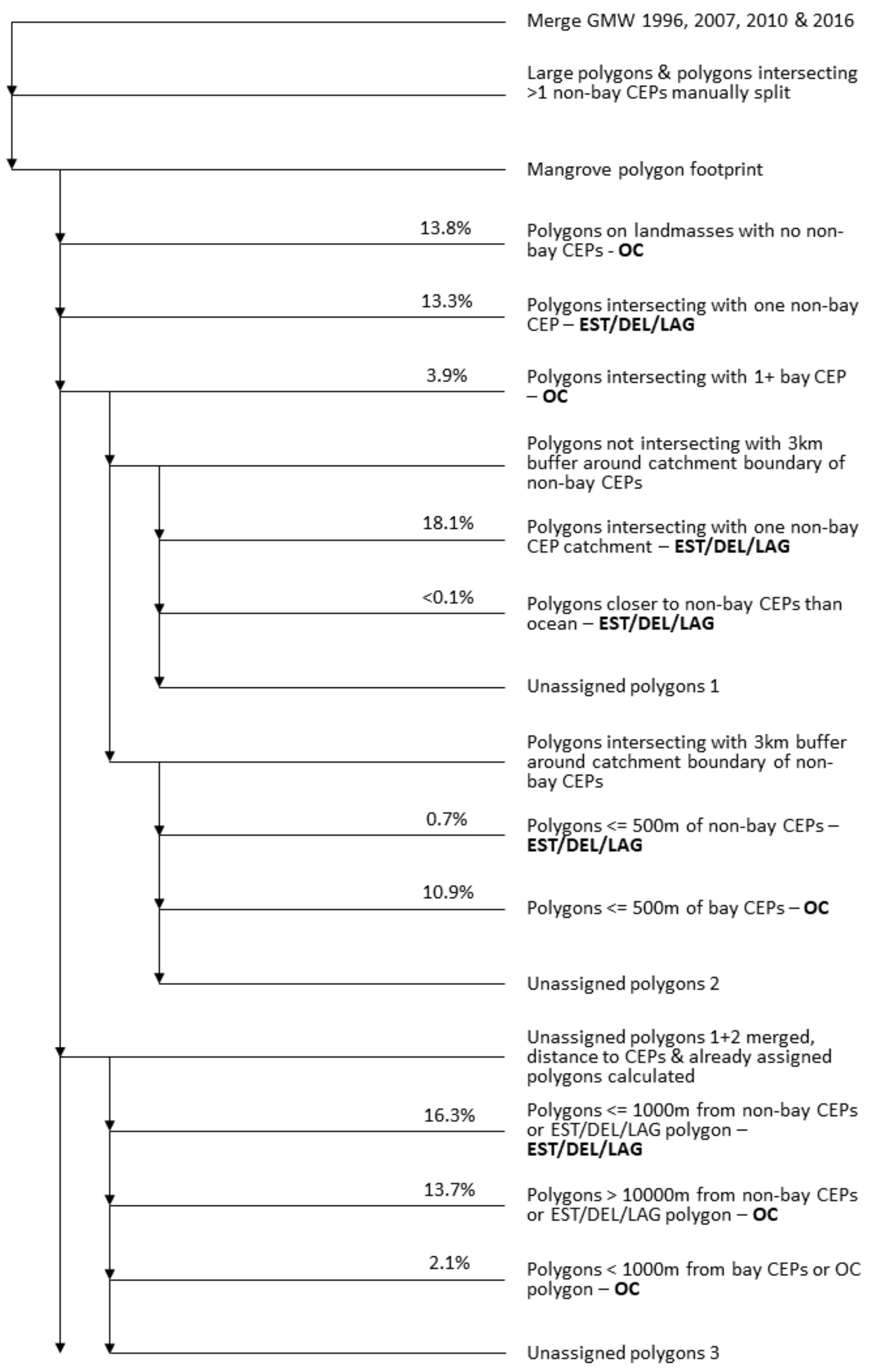

Supplementary Figure 2: Schematic of the stepwise procedure for assigning mangrove patches to a type. Percentages of mangrove patches assigned at each step shown. CEPs = coastal embayment polygons, EST = estuarine, DEL = deltaic, LAG = lagoonal, OC = open coast.

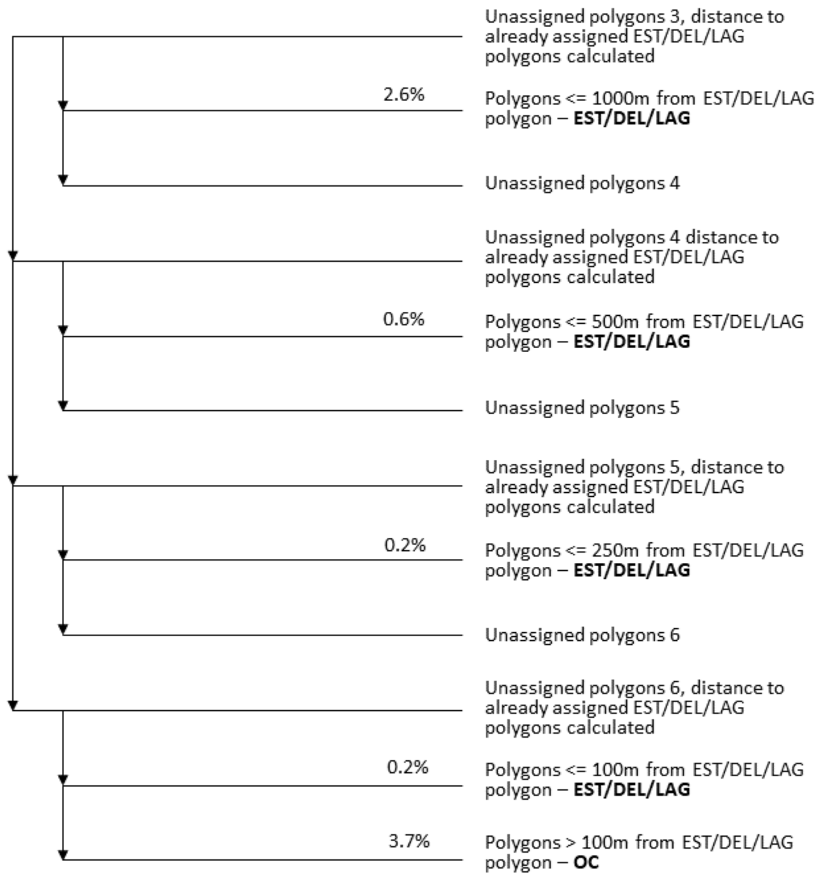

Supplementary Figure 3: Schematic of the stepwise procedure for assigning mangrove patches to a type. Percentages of mangrove patches assigned at each step shown. EST = estuarine, DEL = deltaic, LAG = lagoonal, OC = open coast.

3. Sedimentary Setting

To determine the sedimentary setting of the mangrove typological patches, data on the inorganic suspended particulate matter concentration and tidal amplitude of the site was used. Global inorganic suspended particulate matter concentration data was accessed from the Globcolor website http://www.globcolour.info. The Globcolor project merges outputs from different satellite sensors to improve spatial and temporal coverage[20]. Two hundred and forty individual processed Level-3 files representing monthly global inorganic suspended particulate matter concentration were selected for the period of January 1998 to December 2017 (20 years). These data combine measurements from the SeaWIFS, MERIS, MODIS and VIIRS satellite missions. Across the timeseries the combination of these data sources varied, with data combined using a weighted average approach. Inorganic suspended particulate matter was calculated as the difference between total suspended matter and phytoplankton biomass and is mostly dominated by mineral matter[21]. Data for aquatic areas across the whole globe where available with a pixel resolution of 1/24° (~4.6km at the equator). Monthly inorganic suspended particulate matter data for the period of January 1998 to December 2017 (20 years) were imported into Google Earth Engine[22] and the layers were merged into a 240-image stack. The SPM-OC5 algorithm used to derive inorganic suspended particulate matter concentration computes values ranging between 0 and 100 g/m^3 but is only validated for values less than 50 g/m^3 (The GlobColour Team, pers. comm), therefore values above 50 g/m^3 were truncated. To reduce calculations based on large inland waterbodies a mask was created to remove pixels over land greater 40m in elevation based on the Shuttle Radar Topography Mission 30m Digital Elevation Model[23] and pixels further than 20 km from the Natural Earth coastline (https://www.naturalearthdata.com/downloads/110m-physical-vectors/110m-coastline/). Further

within the image stack, pixels with fewer than 120 records were removed. The mean inorganic suspended particulate matter concentration value of each pixel within the stack was calculated.

Tidal amplitude data, Finite Element Solution tide model, FES2014, were downloaded from AVISO+ products (https://www.aviso.altimetry.fr) with a pixel resolution of 1/16° (~7 km at the equator). A previous iteration (FES2012) was assessed as being one of the most accurate tide models for shallow coastal areas[24], with significant improvements in predictions for those areas identified in FES2014[25]. We selected the principal lunar semi-diurnal or M_2 tidal amplitude for use in our model as it is generally the most dominant tidal constituent[26].

4. Methodological Limitations

As expected of a global scale analysis, methodological limitations exist, related to a) composite settings that may not fit the current typology; and b) classification of geomorphic features at the boundaries between types. One classification identified in other mangrove typologies that is not well captured by our approach is composite settings. Composite settings include high wave energy and high river discharge coasts; where the sediment discharged by the river is subsequently moved by wave action to form a lagoon[3]. Our embayment-based classification did not allow the combination of multiple types. As a result, our lagoon type includes both those fed by small and large estuaries. To classify the coastal embayment polygons, we developed a random forest model which was underpinned by variables describing a polygon's shape, as well as information of the upstream catchment and the amount of precipitation reaching the coast. Visual assessment of the output of the model emphasized issues in separating lagoons and estuaries which required a manual intervention to correct. Further, it was also identified that lagoonal areas had generally been poorly represented by the global coastline used to derive the coastal embayment polygons and therefore manual interventions were again required to reclassify open coast mangrove areas to lagoonal. The use of expert opinion is used widely in conservation science but may introduce bias into decision making[27]. We attempted to minimise such bias by eliciting review of the final designations from a range of researchers.

Difficulties in delineating between geomorphological types is unsurprising as these features represent a continuum between the relative impacts of river, waves, and tides[7]. Existing typologies[4,7,28,29] use the comparative energy inputs from rivers, wave and tide influences to describe different mangrove or estuary types; however, even within individual types e.g.,

deltas[2,30], the relative influences of these drivers produces a range of geometries indicating that at a global scale a single set of shape based descriptors are unlikely to capture such variability.

5. Global Distribution of Mangrove Types

Supplementary Table 5: The maximal extent from the combined GMW 1996, 2007, 2010 and 2016 timesteps of the five largest (km^2) mangrove typological patches in each type

Delta Location	Area (km^2)	Estuary Location	Area (km^2)	Lagoon Location	Area (km^2)	Open Coast Location	Area (km^2)
Niger Delta, Nigeria	6517	Bintuni Bay, Papua, Indonesia	850	Bahia Espiritu Santo, Mexico	888	Papua, Indonesia	907
Deltaic coast of northern Brazil, Brazil	6499	Sembilang National Park, Sumatra, Indonesia	802	Laguna Agua Brava, Mexico	841	Aru Islands, Indonesia	701
Sundarbans, India and Bangladesh	6141	Cacheu River, Guinea Bissau	773	Laguna Celestún, Mexico	793	Tiwi Islands, Australia	509
Deltaic coast of southern Papua, Indonesia	3512	Gabon River, Gabon	731	Southern Florida, USA	766	Aru Islands, Indonesia	391
Deltaic coast of the Gulf of Papua, Papua New Guinea	2087	Casamance River, Senegal	670	Reserva de la Biosfera de Los Petenes, Mexico	723	Carbonate coast of Cuba	375

References

1. Semeniuk, V. & Semeniuk, C. Deltas. in *Encyclopedia of Estuaries* (ed. Kennish, M. J.) 177–186 (Springer Dordrecht, 2016).
2. Bhattacharya, J. P. Deltas. in *Facies Models Revisited* (eds. Posamentier, H. W. & Walker, R. G.) 237–292 (SEPM Society for Sedimentary Geology, 2006).
3. Thom, B. G. Coastal landforms and geomorphic processes. in *The Mangrove Ecosystem: Research Methods* (eds. Snedaker, S. C. & Snedaker, J. G.) 18–35 (UNESCO, 1984).
4. Woodroffe, C. Mangrove sediments and geomorphology. in *Tropical Mangrove Ecosystems* (eds. Robertson, A. I. & Alongi, D. M.) 7–41 (American Geophysical Union, 1992).
5. Pritchard, D. W. What is an estuary: Physical standpoint. in *Estuaries* (ed. Lauff, G. H.) 3–5 (American Association for the Advancement of Science, Publication 83, 1967).
6. Kennish, M. J. Environmental threats and environmental future of estuaries. *Environ. Conserv.* **29**, 78–107 (2002).
7. Twilley, R. R. & Rivera-Monroy, V. H. Ecogeomorphic models of nutrient biogeochemistry for mangrove wetlands. in *Coastal Wetlands: An Integrated Ecosystem Approach* (eds. Perillo, G. M. E., Wolanski, E., Cahoon, D. R. & Brinson, M. M.) 641–684 (Elsevier, 2009).
8. Woodroffe, C. D. *et al.* Mangrove sedimentation and response to relative sea-level rise. *Ann. Rev. Mar. Sci.* **8**, 243–266 (2016).
9. Kjerfve, B. Coastal lagoons. in *Coastal Lagoon Processes* (ed. Kjerfve, B.) 1–8 (Elsevier, 1994).
10. Chapman, P. M. Management of coastal lagoons under climate change. *Estuar. Coast.*

Shelf Sci. **110**, 32–35 (2012).

11. OzCoasts. Embayments and drowned river valleys. (2018). Available at: https://ozcoasts.org.au/conceptual-diagrams/science-models/geomorphic/emb/. (Accessed: 20th November 2018)
12. Giri, C. *et al.* Status and distribution of mangrove forests of the world using earth observation satellite data. *Glob. Ecol. Biogeogr.* **20**, 154–159 (2011).
13. Spalding, M. D., Kainumu, M. & Collins, L. *World Atlas of Mangroves*. (Earthscan, 2010).
14. Huh, O. K., Coleman, J. M., Braud, D. & Kiage, L. *World Deltas Database. Appendix A. The Major River Deltas Of The World. Report*. (2004).
15. Coleman, J. M. & Huh, O. K. *Major World Deltas: A Perspective From Space*. (2003).
16. Lehner, B., Verdin, K. & Jarvis, A. New global hydrography derived from spaceborne elevation data. *Eos, Trans. Am. Geophys. Union* **89**, 93–94 (2008).
17. Domisch, S., Amatulli, G. & Jetz, W. Near-global freshwater-specific environmental variables for biodiversity analyses in 1 km resolution. *Sci. Data* **2**, 150073 (2015).
18. R Core Team. R: A language and environment for statistical computing. (2019).
19. Olson, D. M. *et al.* Terrestrial Ecoregions of the World: A new map of life on earth. *BioScience* **51**, 933–938 (2001).
20. ACRI-ST GlobColour Team. *Globcolour. Product User's Guide version 4.1*. (2017).
21. Gohin, F. Annual cycles of chlorophyll-a, non-algal suspended particulate matter, and turbidity observed from space and in-situ in coastal waters. *Ocean Sci.* **7**, 705–732 (2011).
22. Gorelick, N. *et al.* Google Earth Engine: Planetary-scale geospatial analysis for everyone. *Remote Sens. Environ.* **202**, 18–27 (2017).

23. Farr, T. G. *et al.* The Shuttle Radar Topography Mission. *Rev. Geophys.* **45**, RG2004 (2007).

24. Stammer, D. *et al.* Accuracy assessment of global barotropic ocean tide models. *Rev. Geophys.* **52**, 243–282 (2014).

25. Carrère, L., Lyard, F., Cancet, M. & Guillot, M. FES 2014, a new tidal model on the global ocean with enhanced accuracy in shallow seas and in the Arctic region. in *EGU General Assembly 2015, held 12-17 April, 2015 in Vienna, Austria* (2015).

26. Ray, R. D., Eanes, R. J., Egbert, G. D. & Pavlis, N. K. Error spectrum for the global M_2 ocean tide. *Geophys. Res. Lett.* **28**, 21–24 (2001).

27. Martin, T. G. *et al.* Eliciting expert knowledge in conservation science. *Conserv. Biol.* **26**, 29–38 (2012).

28. Dalrymple, R. W., Zaitlin, B. A. & Boyd, R. A conceptual model of estuarine sedimentation. *J. Sediment. Petrol.* **62**, 1130–1146 (1992).

29. Ewel, K. C., Twilley, R. R. & Ong, J. Different kinds of mangrove forests provide different goods and services. *Glob. Ecol. Biogeogr. Lett.* **7**, 83–94 (1998).

30. Syvitski, J. P. M. & Saito, Y. Morphodynamics of deltas under the influence of humans. *Glob. Planet. Change* **57**, 261–282 (2007).
